# Point of Care Ultrasound as a Key Tool in the Evaluation of a Woman with Syncope

**DOI:** 10.24908/pocus.v9i1.17329

**Published:** 2024-04-22

**Authors:** Katherine Guío Rodríguez, Jenny Del Pilar Rico Mendoza, Elkin René Barrios Peralta

**Affiliations:** 1 Emergency Medicine Resident, Department of Internal Medicine, Pontificia Universidad Javeriana Bogotá Colombia; 2 Cardiology Fellow, Department of Internal Medicine, Pontificia Universidad Javeriana Bogotá Colombia; 3 Specialization in Emergency Medicine, Department of Internal Medicine, Pontificia Universidad Javeriana Bogotá Colombia

**Keywords:** Syncope, Bedside Ultrasonography, Emergency Department, Intensive Care, Cardiac Neoplasms

## Abstract

Using point of care ultrasound (POCUS) to evaluate patients with syncope in the emergency department facilitates the timely diagnosis of life-threatening pathologies. Case: A 56-year-old woman presented to the emergency department of a hospital in Bogotá, Colombia, for a syncopal episode. Vital signs, physical examination, electrocardiogram, and routine laboratory tests were normal. Cardiac POCUS was performed, which identified an echogenic mass located in the left atrium, measuring 35x28mm, which in left atrial systole appeared to occupy the entire chamber. She underwent surgical resection of the mass and histopathology revealed atrial myxoma. Conclusions: POCUS was useful in the rapid diagnosis of atrial myxoma in a woman presenting with syncope.

## Introduction

Syncope is a frequent reason for consultation in clinical practice. Syncope has a prevalence of approximately 40% and accounts for 1-3% of emergency department visits and 6% of hospital admissions 1, 2. The causes can be divided into cardiovascular, cerebrovascular, vascular tone, blood flow disorders, and others that mimic syncope. The most relevant causes in this latter group are seizures, metabolic events (hypoglycaemia, hypoxia, symptomatic anaemia), and psychogenic 3. It has been estimated that syncope has a recurrence rate of 13.5%, of which 6-30% of these involve cardiac causes. Cardiovascular causes of syncope include cardiac neoplasms 4. Primary intracardiac tumours occur infrequently with an incidence of 1.38-30 per 100 000 persons per year. In comparison, secondary cardiac tumours are 20-30 times more common 5. Most primary cardiac neoplasms are benign tumours (75%) and within this group the most frequent, although rare, is atrial myxoma. Myxoma is more common in women (65-70%), and although it can be found in any age group, its peak incidence is between the fourth and sixth decade of life 6. Its clinical presentation is highly variable in relation to its embolic and obstructive effects, as well as its location, size, and mobility 7. In addition to syncope, myxoma can manifest as systemic embolism, heart failure, or even sudden cardiac death 8.

Point of care ultrasound (POCUS) has become an essential tool for physicians in the emergency department, enabling faster patient care and serving as a guide for performing procedures and making therapeutic decisions. POCUS can be used to identify life-threatening pathologies in which timely detection can have an impact on reducing mortality 9.

In the emergency department, POCUS has become a fundamental tool for patient evaluation. In this particular case it was very useful for the diagnosis of atrial myxoma, which was the basis for rapid decision-making in a patient who presented with syncope.

## Case

A 56-year-old woman with no significant medical history presented to the emergency department for an episode of loss of consciousness with loss of postural tone lasting approximately 60 seconds with complete recovery. Syncope was preceded by chest pain, but not associated with dizziness, sensation of heat, sweating, nausea, vomiting, or related to changes in position or exertion. She reported that she had been suffering for one week from oppressive chest pain in the left hemithorax with irradiation to the left upper limb that was self-limiting, intermittent, not associated with exertion or stress, and exacerbated before loss of consciousness.

On initial assessment by the emergency department, the patient had normal vital signs, no signs of acute respiratory distress, normal cardiopulmonary auscultation, no carotid bruit, soft abdomen with no signs of peritoneal irritation, no peripheral edema, preserved distal perfusion, and no focal neurological findings.

The initial electrocardiogram documented sinus rhythm, a heart rate of 67 bpm, normal axis, no signs of chamber enlargement, no atrioventricular conduction disturbances or intraventricular block, ST without alterations, and QTc 419 ms. Troponin was negative, and the laboratory exam was within normal limits. The chest x-ray was also normal. 

**Figure 1:**
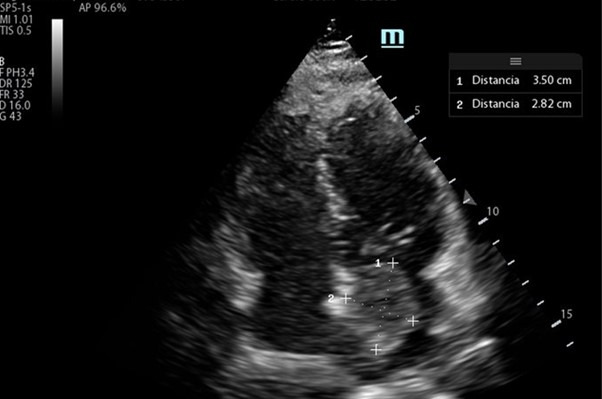
Apical 4 chamber view. Mass in left atrium.

Given that the patient had no history of structural heart disease or coronary artery disease, her examination in the emergency department was normal and her six-hour observation stay was asymptomatic, hospital discharge was considered for outpatient referral to transthoracic echocardiography. However, cardiac POCUS revealed a 35x28mm echogenic mass in the left atrium that occupied the entire chamber in left atrial systole. There was no pericardial effusion. E-point septal separation (EPSS) was 4mm with adequate global contractility of the left ventricle, normal right ventricle/left ventricle ratio, and aortic root of 33mm without dissection flap (Figure 1, Video S1). 

Due to the large left atrial mass, discharge was cancelled and the patient was assessed by the cardiology department. They performed a transthoracic echocardiogram and found a slightly dilated left atrium of 34 ml/m2 occupied by a heterogeneous mass of 33x34mm with well-defined borders. This occupied more than 50% of the atrial area and adhered to the interatrial septum without compromising the mobility of the mitral valve, as suggestive of myxoma. The left ventricle was of normal size and shape, had mild eccentric hypertrophy, and a left ventricular ejection fraction (LVEF) of 61% with signs of type II diastolic dysfunction.

The patient was referred to cardiovascular surgery, who considered her to be a candidate for urgent surgery due to the size of the atrial lesion and the high risk of obstruction of the left ventricular inflow tract as a generator of embolism and sudden death. Left heart catheterization was performed and the coronary arteries were found to be angiographically normal without significant stenosis. The open excision procedure to remove the cardiac tumour was performed. Intraoperative findings were a 4x4cm left atrial mass with a 2cm pedicle, adhered to the interatrial septum and the posterior wall. Resection was performed from its pedicle without macro residues adhering to the wall and without rupture of the septum. The procedure was uncomplicated and the patient was monitored in the intensive care unit in the postoperative period with adequate recovery. For this reason she was transferred to general hospitalisation with comprehensive cardiovascular rehabilitation therapy. On the fifth postoperative day, the patient was discharged from the hospital. Pathology study confirmed the diagnosis of cardiac atrial myxoma.

## Discussion

POCUS is a non-invasive diagnostic tool that has been gaining popularity in the emergency department due to the significant value it provides in decision-making. POCUS enables early narrowing of the differential diagnoses and can guide decisions regarding adjunctive testing while facilitating faster treatment initiation. The benefits of using POCUS have been demonstrated in a wide variety of clinical conditions such as undifferentiated shock, cardiac arrest, trauma, chest pain, dyspnoea, syncope, and abdominal pain, among others 9.

Concerning syncope, different methods have been described for clinical decision-making in the emergency department for risk stratification based on clinical history, physical examination, and electrocardiographic findings. However, none of these methods can be widely used and are not superior to clinical judgement in predicting short-term adverse outcomes 10.

As previously mentioned, although infrequent, myxoma can be a cause of syncope. The clinical presentation varies widely from an asymptomatic incidental mass to severe, life-threatening cardiovascular complications, depending on location, size, and mobility. There is a classic triad that raises diagnostic suspicion consisting of intracardiac obstruction, embolization and constitutional symptoms. Despite the diverse clinical presentations, POCUS may be beneficial in suspected cases in the emergency department 11.

It is the primary task of the emergency specialist to identify patients with high-risk cardiovascular disease requiring urgent testing with hospital admission. Patients presenting with high-risk syncope are most likely to have presented with syncope of cardiac origin. Structural heart disease and primary electrical disorders are the most important risk factors for sudden death and total mortality in patients with syncope 3. Within the admission history, clinical features such as new presentation of chest pain, dyspnoea, abdominal pain or headache, syncope on exertion, in supination or preceded by sudden onset palpitations guide the clinician in suspecting that the patient may be presenting with high-risk syncope.

A single-centre prospective observational cohort study conducted at the Hospital of "Città della Salute e della Scienza di Torino", Turin, Italy tested the accuracy of the integrated POCUS approach with clinical assessment and electrocardiographic findings in risk-stratifying patients in the emergency department. They included patients who had presented with a history of syncope in whom the etiology had not been identified despite a structured approach with clinical history, physical examination, and electrocardiogram that classified them in neither high nor low-risk group (NHNL) 12. This increased diagnostic accuracy by approximately 10% and reduced risk categorisation errors by 4% after including the integrated POCUS approach to clinical assessment 12.

In the case of our patient, she was suffering from high-risk syncope because of the presentation associated with chest pain. POCUS was the key diagnostic tool in identifying structural cardiac pathology in this case by visualising the presence of an atrial mass with a high risk of mechanical obstruction and sudden death. Without its use, diagnosis would have been delayed, increasing the risk of complications.

## Conclusions

Syncope is a frequent reason for consultation with the emergency department. Its causes may be multiple, including cardiovascular causes that can be life-threatening. Implementation of POCUS with clinical assessment in the ED can increase diagnostic accuracy in the work-up of a patient with syncope, helping to identify patients at high risk of adverse events in the short term. Emergency providers should be trained in POCUS and be able to identify life-threatening etiologies.

## Disclosures

 The authors report no conflicts of interest or funding sources.

## Patient Consent

Consent to publish this case was obtained from the patient via an informed consent form. 

## Supplementary Material

 Video S1 Apical 4 chamber view. Mass in left atrium.
